# Inflammatory Factors Induce Thrombosis through the miR-146b-3p/p38MAPK/COX-2 Pathway

**DOI:** 10.1155/2020/8718321

**Published:** 2020-04-01

**Authors:** Zhengjia Su, Fang Wu

**Affiliations:** Department of Geratology, Ruijin Hospital, Shanghai Jiao Tong University School of Medicine, Shanghai, China

## Abstract

**Objective:**

Inflammatory responses play important roles in the pathogenesis of atherosclerosis. The purpose of this study was to investigate the relationship between microRNA-146b-3p (miR-146b-3p) and inflammatory factors in thrombosis.

**Method:**

THP-1 cells were cultured in vitro, Western blot was used to determine the protein levels of COX-2 and p38MAPK in the cells, and real-time PCR was used to detect the mRNA expression of miRNA-146b-3p and COX-2. A lentiviral expression vector of miRNA-146b-3p and its inhibitor were constructed to transfect THP-1 cells. COX-2 and p38MAPK expression in transfected cells was detected by Western blot and real-time PCR, respectively.

**Results:**

Ang II and TNF-*α* could elevate the expression of COX-2 in monocytes. The expression of COX-2 was upregulated by p38MAPK, which could be phosphorylated by Ang II, while there was an increasing tendency of p38MAPK phosphorylation after TNF-*α* stimulation. In addition, COX-2 expression and P38MAPK phosphorylation could be downregulated by miRNA-146b-3p and upregulated by the miRNA-146b-3p inhibitor. Ang II could increase miR-146b-3p expression, although there was no significant difference; however, the expression of miR-146b-3p was enhanced significantly by TNF-*α*.

**Conclusion:**

Our data implied that altered expression of miR-146b-3p was closely related to the progression of inflammation mediating the P38MAPK/COX-2 pathway. We suggest that the miR-146b-3p/p38MAPK/COX-2 pathway plays a key role in inflammation and arterial thrombosis.

## 1. Introduction

Atherosclerosis (AS) is a common pathological process during arterial thrombosis. Inflammation plays an important role in the pathogenesis of atherosclerosis [1]. Excessive inflammatory response of vascular cells, especially monocytes and macrophages, is considered a pivotal pathogenesis mechanism underlying atherogenesis. Mounting evidence suggests that cytokines like tumor necrosis factor-*α* (TNF-*α*) and angiotensin-II (Ang II) are increased in human arterial thrombosis [2, 3]. Cyclooxygenase-2 (COX-2), a proinflammatory mediator, has been found to be increased under the stimulation of Ang II and TNF-*α*. It is involved in the pathogenesis of a variety of inflammatory-related diseases including AS [4]. However, the contribution and regulation of various cytokines and signal transduction pathways in inflammation-mediated AS development remain undefined by far.

MicroRNA (miRNA) is a class of endogenous, small-molecule, noncoding, and highly conserved single-stranded RNAs, which is important in the regulation of gene expression. It binds to the 3′nontranscribed region (3′-UTR) of the mRNA to promote mRNA degradation, inhibits its transcription, and regulates posttranscriptional gene expression [5]. Multiple miRNAs are involved in the regulation of inflammatory reactions and contribute to the progression of AS [6]. Previous reports demonstrated that miR-146b-3p was closely related to the progression and development of inflammation and arterial thrombosis [7]. Mitogen-activated protein kinase (MAPK) which is a potential target of multiple miRNAs may promote the formation of arterial thrombosis by regulating the function of miRNA in the process of arterial thrombosis. However, it is still unclear how miR-146b-3p participates in the pathology inflammatory factor-induced thrombosis.

In this study, we focused on the role of miR-146b-3p in the development and progression of thrombosis. We aimed to investigate the relationship between the miR-146b-3p/p38MAPK/COX-2 pathway and inflammatory factors including TNF-*α* and Ang II and to clarify its molecular mechanism.

## 2. Materials and Methods

### 2.1. Cell Culture

The 293T cells were bought from JIKAI Company (Shanghai, China) and grown in Dulbecco's modified Eagle's medium (DMEM) containing 10% fetal bovine serum (Gibco). THP-1 cells were provided by the Shanghai Chinese Academy of Sciences Health Science Research Center and cultured in RPMI 1640 medium containing 10% fetal bovine serum, 1% penicillin-streptomycin solution, and 1% Max (Gibco). Both kinds of cells were incubated at 5% CO_2_ at 37°C.

All the experiments were repeated at least thrice independently in this study.

### 2.2. Construction of Plasmids

A lentiviral GV369 vector was purchased from JIKAI Company (Shanghai, China). miRNA-146b sequences were amplified by PCR and cloned into EcoRI and Age I sites of the vector, the recombinant plasmid named pGV369-146b. The presence of a target gene was confirmed by sequence analysis.

### 2.3. Lentivirus Packaging

To generate lentivirus from the recombinant plasmids, 293T cells were grown in a cell culture plate and cultured with serum-free medium for 2 hours when the cell density reached 70% to 80%. Then, the cells were transfected with 20 *μ*g of the recombinant plasmid pGV369-146b together with 15 *μ*g of the helper plasmid PHelper 1.0 vector plasmid, and 10 *μ*g of the pHelper 2.0 vector plasmid. At 6 h posttransfection, the cells were washed with phosphate-buffered saline (PBS) and added with DMEM containing 10% FBS; then, the supernatant of 293T cells was collected 48 hours after transfection, and the harvested virus was named L-GV369-146b. The supernatant was filtered through a 0.45 *μ*m filter and centrifuged at 25000 rpm for 2 hours. The supernatant was discarded, and the virus was resuspended with virus preservation solution. Finally, the virus solution was centrifuged at 10000 rpm for 5 minutes, and the supernatant was stored.

### 2.4. Lentivirus Infection

Five groups included a conventional culture (CM) group, CM plus 5 *μ*g/ml polybrene group, Eni.S group, Eni.S plus 5 *μ*g/ml polybrene group, and blank control group. Each group included Pfu 1 × 10^8^ TU/ml, 1 × 10^7^ TU/ml, and 1 × 10^6^ TU/ml of virus-infected cells, respectively. The THP-1 cells were seeded on a 12-well plate with 1 × 10^6^/well for mRNA expression detection and incubated in a 6 cm dish at 3 × 10^6^/well to detect protein expression. The results of preexperiment showed that THP-1 cells were best infected in condition of MOI 100, Eni.S plus 5 *μ*g/ml polybrene; therefore, THP-1 cells were infected according to the above conditions.

### 2.5. Western Blot

The cells were scraped and transferred to an EP tube, then centrifuged at 5000 rpm/min for 5 minutes. The samples were collected and extracted by RIPA buffer (Thermo) on ice for 30 min. Then, the BCA Kit (Bioteke) was used to determine the protein concentration. Equal amounts of protein were analyzed by Western blotting, and GAPDH was used to determine the amounts of protein from the infected cells. The proteins were subjected to SDS-10% PAGE, followed by transferring to PVDF membranes. The membranes were blocked with 5% skimmed milk and then were incubated with anti-COX-2, p38MAPK, p-p38MAPK, and anti-GAPDH primary antibodies, followed by incubation with horseradish peroxidase- (HRP-) conjugated antibodies to detect COX-2, p38MAPK, p-p38MAPK, and GRAPDH binding, respectively.

### 2.6. Real-Time PCR Analysis for RNA Expression

Total RNA was extracted with TRIzol (Invitrogen) according to the manufacturer's protocol. Real-time PCR was performed using Fast Start Universal SYBR Green Master (Roche). Glyceraldehyde-3-phosphate dehydrogenase (GAPDH) was used as an endogenous control. Fold changes in the mRNA expression level normalized to GAPDH were calculated using the -*ΔΔ*CT method. The following primers were used: COX-2-RT-P1, 5′-CCCACCCATGTCAAAACCGA-3′; COX-2-RT-P2, 5′-CCGGGTACAATCGCACTTATACT-3′; H-GAPDH-P1, 5′-ATGGGGAAGGTGAAGGTCG-3′; H-GAPDH-P2, 5′-GGGGTCATTGATGGCAACAATA-3′; miR-146b-P1, 5′-CCUGGCACUGAGAACUGAAUUCCAUAGGCUGUGAGCUCU-3′; and miR-146b-P2, 5′-AGCAAUGCCCUGUGGACUCAGUUCUGGUGCCCGG-3′.

### 2.7. Statistical Analysis

Numerical data were reported as the mean ± SD. The GraphPad Prism version 5 software program was used for statistical analyses. Continuous variables were analyzed by one-way ANOVA or *T*-test to compare differences among the groups. Categorical data were analyzed by the chi-square test. *P* < 0.05 was considered significant.

## 3. Results

### 3.1. COX-2 Expression Was Induced by Ang II and TNF-*α* in THP-1 Cells

Two inflammatory factors including Ang II and TNF-*α* were applied to stimulate the human monocytic cell line THP-1 cells *in vitro*. By RT-PCR and Western blot analysis, we found that COX-2, a key gene which is widely reported to be responsible for the inflammation process in thrombosis, was significantly upregulated in THP-1 cells after the treatment of Ang II (100 nmol/l) or TNF-*α* (10 ng/ml).

Consistent with previous results [8], both the mRNA expression of COX-2 in the cells treated with Ang II and that in the cells treated with TNF-*α* were significantly increased after 30 minutes. The protein expression of COX-2 was significantly increased after 1 hour in those 2 groups.

**Figure 1:**
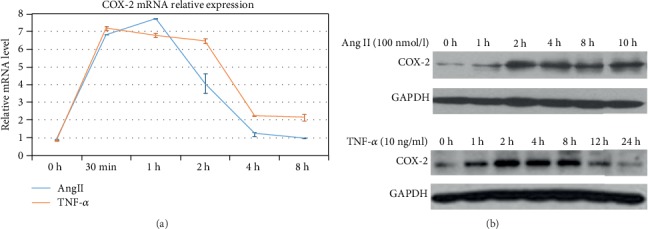
COX-2 expression was induced by Ang II and TNF-*α* at different time points: (a) the RT-PCR assay was used to detect COX-2 mRNA expression; (b) the Western blot assay was used to detect COX-2 protein expression.

COX-2 mRNA peaked at 60 min after Ang II treatment, while it peaked at 30 min after TNF-*α* treatment (7.749 ± 0.009, 7.202 ± 0.120, *P* < 0.05) (Figure 1(a)). COX-2 protein level reached its peak value at 2 h after Ang II treatment while it peaked at 4 h after TNF-*α* treatment (Figure 1(b)). The results showed that treatment of these two factors could induce the expression of COX-2 in THP-1 cells.

### 3.2. Ang II and TNF-*α* Induce p38MAPK Phosphorylation in THP-1 Cells

**Figure 2:**
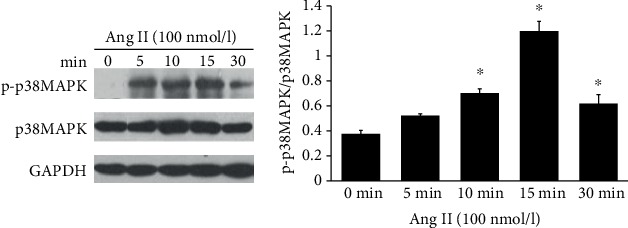
Ang II induced the expression of phosphorylation of p38MAPK in THP-1 cell. ^∗^*P* < 0.05. The bands at 2 min were cropped in the Western blot result to show the phosphorylation change of p38MAPK at 5 min, 10 min, 15 min, and 30 min, which was consistent with the TNF-*α* treatment time.

To further validate the activation of THP-1 cells, phosphorylation of p38MAPK in THP-1 cells was also measured. We treated the cells with Ang II and TNF-*α* separately and detected the effect of p38MAPK phosphorylation at 5 min, 10 min, 15 min, and 30 min. The data showed that phosphorylation of p38MAPK could be significantly activated after treatment with Ang II for 10 minutes, and p-p38MAPK expression reached maximum after induction for 15 minutes. The results demonstrated that Ang II could significantly induce the phosphorylation of p38MAPK in THP-1 cells (*P* < 0.05) (Figure 2).

**Figure 3:**
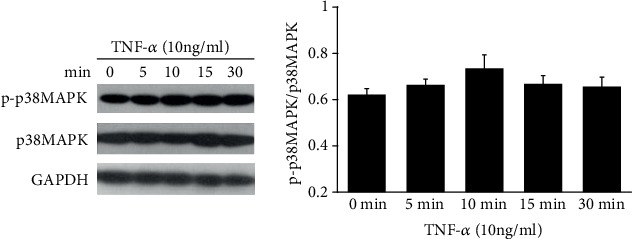
Expression of phosphorylation of p38MAPK was assessed in TNF-*α*-induced THP-1 cells.

The expression of p-p38MAPK increased after TNF-*α* stimulation, and it reached maximum after induction of 10-minute treatment, which indicated that TNF-*α* was better able to promote p38MAPK phosphorylation in THP-1 cells, though there was no statistical difference before and after TNF-*α* treatment (Figure 3).

### 3.3. Ang II Induces COX-2 Expression through p38MAPK in THP-1 Cells

**Figure 4:**
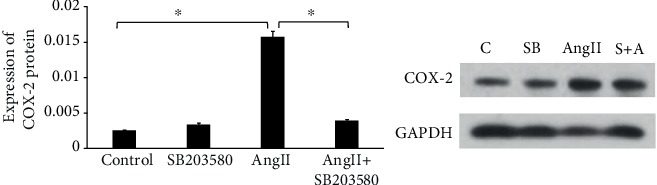
The effect of SB20358 on COX-2 protein expression induced by Ang II in THP-1 cells. ^∗^*P* < 0.05. COX-2 expression was significantly increased by Ang II treatment compared with control. COX-2 expression was significantly decreased in the Ang II+SB203580 group compared with the Ang II group.

We added the p38MAPK inhibitor SB203580 (25 nmol/l) into the cell culture medium and then treated the THP-1 cells with Ang II for 2 hours. COX-2 protein level in THP-1 cells treated with SB203580 was significantly downregulated (*P* < 0.05) (Figure 4), indicating that Ang II might induce COX-2 expression through p38MAPK in THP-1 cells.

### 3.4. Ang II and TNF-*α* Could Induce the Expression of miR-146b-3p in THP-1 Cells

**Figure 5:**
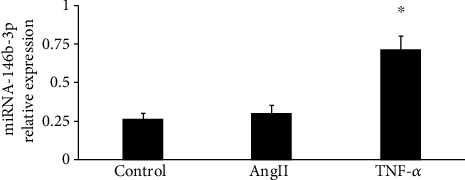
The expression of miRNA-146-3p induced by Ang II and TNF-*α* in THP-1 cells. ^∗^*P* < 0.05.

To further reveal the role of miR-146b-3p in monocyte activation, Ang II and TNF-*α* were added into THP-1 cell culture medium, and miR-146b-3p expression was detected. We treated THP-1 cells with Ang II for 2 hours and TNF-*α* for 4 hours. The expression of miR-146b-3p was significantly upregulated after TNF-*α* treatment (0.267 ± 0.036 vs. 0.720 ± 0.086, *P* < 0.05). With Ang II treatment, the expression of miR-146b-3p showed an increasing tendency, although there was no significant difference (0.267 ± 0.036 vs. 0.304 ± 0.053, *P* > 0.05) (Figure 5).

### 3.5. Effects of miRNA-146b-3p on p38MAPK Phosphorylation and COX-2 Expression

**Figure 6:**
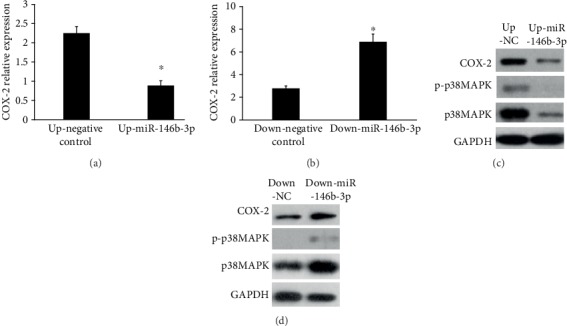
Effects of miRNA-146b-3p on COX-2 expression and p38MAPK phosphorylation. (a) COX-2 mRNA expression at 24 h after transfection with miR-146b-3p. (b) COX-2 mRNA expression at 24 h after transfection with the miR-146b-3p inhibitor. (c) Influences on p38MAPK phosphorylation and COX-2 protein expression after transfection with miR-146b-3p. (d) Influences on p38MAPK phosphorylation and COX-2 protein expression after transfection with the miR-146b-3p inhibitor. ^∗^*P* < 0.05.

To clarify the relationship between miRNA-146b-3p and p38MAPK phosphorylation, as well as the relationship between miRNA-146b-3p and COX-2 expression, cells were transfected with the miRNA-146b-3p or miRNA-146b-3p inhibitor. THP-1 cells were infected with lentivirus at MOI of 100; then, we measured COX-2 mRNA expression levels at 24 h after transfection. We found that COX-2 expression decreased significantly after transfection with miR-146b-3p (2.251 ± 0.171, 0.885 ± 0.128, *P* < 0.05) (Figure 6(a)), while transfection with the miR-146b-3p inhibitor resulted in high expression of COX-2 (2.785 ± 0.210, 6.885 ± 0.695, *P* < 0.05) (Figure 6(b)).

Phosphorylation of P38MAPK was in accordance with COX-2 expression, which was apparently downregulated in miRNA-146b-3p-transfected cells (Figure 6(c)) and upregulated significantly in the inhibitor group (Figure 6(d)) compared with negative control group.

## 4. Discussion

COX-2, an important rate-limiting enzyme in the process of arachidonic acid metabolism, affects the production of prostaglandin and TXA-2 and has a close correlation with atherosclerotic formation. Previous reports showed that COX-2 was regulated by a variety of inflammatory cytokines that might regulate arterial thrombosis [9, 10]. In this study, we also found that inflammatory factors like Ang II and TNF-*α* could increase COX-2 expression in THP-1 cells. Both the mRNA expression of COX-2 in the cells treated with Ang II and that in the cells treated with TNF-*α* were significantly increased after 30 minutes. The protein expression of COX-2 was significantly increased after 1 hour by the treatment with those 2 factors. Interestingly, COX-2 protein level reached its peak at 2 h, and COX-2 mRNA expression peaked at 1 h after Ang II treatment. Furthermore, when the cells were exposed to TNF-*α*, the protein expression of COX-2 reached the peak in 4 hours, while COX-2 mRNA expression which was still significantly higher at 4 h reached the peak after 30 minutes. The mRNA and protein expression of COX-2 did not reach the peak synchronously, although the time at which it started to increase remarkably was very close. This might be owing to experimental conditions, detection methods, and different half-lives. mRNAs are highly degradable and become stable when translated into proteins.

However, our data indicated that expression of COX-2 was regulated by Ang II and TNF-*α* and changed with time, which was consistent with previous reports [11]. But the mechanism of inflammation-induced COX-2 elevation is still unclear.

MAPKs contain a series of evolutionarily conserved proteins with the Ser/Thr kinase domain; among which, p38MAPK plays a wide range of functions in various biological processes including apoptosis, pathogen infection, and cell differentiation. Thrombosis is a chronic inflammatory process, and it was also reported that p38MAPK triggered inflammatory response [12]. Therefore, in the present study, we treated THP-1 cells with Ang II and TNF-*α*, respectively, to observe p38MAPK phosphorylation. The results showed that p38MAPK phosphorylation could be induced by Ang II, which was consistent with previous studies [12]. We found that p38MAPK phosphorylation was not significantly increased after stimulation with TNF-*α*, although there was an increasing tendency of p38MAPK phosphorylation, which might be related to cell types and experimental conditions, and further studies are needed. Then, we added the p38MAPK inhibitor SB203580 into Ang II-treated cells, and it showed that SB203580 could decrease COX-2 expression. In this regard, we supposed that Ang II might enhance the expression of COX-2 partly through the p38MAPK pathway.

miRNA, a family of conservative and small noncoding RNA, regulates gene expression by splicing target genes or inhibiting translation. It is demonstrated that miRNA modulates plenty of biological processes, including vascular smooth muscle cell proliferation and migration, monocyte adhesion and its differentiation to macrophages, maintenance of vascular integrity, and cholesterol metabolism [13–15]. miR-146 is a typical multifunctional miRNA, which regulates different target genes in the same pathway. Its target genes are involved in the occurrence and development of multiple physiological and pathological processes like immune response, inflammation, hematopoiesis, and tumor growth. Vasa-Nicotera et al. found that decreased miRNA-146 could lead to premature senescence of human umbilical vein endothelial cells [16]. Studies have shown that miR-146 expression in peripheral blood monocytes decreased significantly while TRAF-6 mRNA expression increased in patients with type 2 diabetes mellitus and miR-146 expression is negatively linked to TRAF-6 and NF-*κ*B mRNA levels as well as circulating TNF-*α* and IL-6 [17]. Furthermore, it has been demonstrated that the expression of miR-146b-3p decreased significantly in thrombosis patients [18]. Therefore, in the present study, miRNA-146b-3p was chosen as an object and its effect on inflammation and thrombosis was worthy of study.

In our study, compared with control groups, the expression of miR-146b-3p was significantly increased in TNF-*α*-induced groups. An increasing tendency could also be observed in Ang II-induced groups, although there was no significant difference. Further experiments are needed to detect miR-146b-3p expression at different time points after Ang II treatment to illustrate the relation between Ang II and miR-146b-3p. The present result indicated that miR-146b-3p and inflammatory factors were closely related. And we attempted to find out whether miR-146b-3p affected the expression of COX-2 or p38MAPK. THP-1 cells were then transfected with miRNA-146b-3p overexpression and inhibitor in the study. We found that COX-2 expression and P38MAPK phosphorylation were apparently downregulated in the cells transfected with miRNA-146b-3p and upregulated significantly in the inhibitor group compared with the negative control group. The results indicated that miR-146b-3p could downregulate COX-2 expression and p38MAPK phosphorylation. Interestingly, we also found that Ang II and TNF-*α* could increase the expression of COX-2 and miRNA-146b-3p, as well as p38MAPK phosphorylation, suggesting that miRNA-146b-3p might play an important negative feedback regulating role in inflammation-mediated thrombosis.

Recent studies show that JAK-STAT plays a very important role in the course of atherosclerosis [19]. Animal experiment revealed the activation of the STAT3 pathway in atherosclerotic disease [20]. miR-146 inhibits the expression of IL-6 through the NF-*κ*B pathway, leading to the reduction of STAT3 phosphorylation and the formation of negative feedback [21]. Walker et al. found that miR-146b is a target gene of STAT3, but meanwhile, it also provides negative feedback to STAT3 [22]. It is found that the STAT3 signaling pathway mediated by IL-10 can increase the activity of miR-146 [23]. Overexpression of miR-146 can downregulate IL-6 and TNF-*α* expression in dendritic cells [24]. In this study, the expression of miR-146b-3p increased significantly after TNF-*α* treatment, which was consistent with previous studies. Meanwhile, increased miR-146b-3p was involved in the process of thrombotic diseases through the MAPK pathway to regulate the expression of the downstream COX-2 gene. Recent reports have shown that overexpression of miR-146 could block the activation of the endothelial system and inhibit NF-*κ*B, AP-1, and MAPK/EGR signaling pathways. Correspondingly, NF-*κ*B and MAPK can inhibit the expression of miR-146a/b feedback, forming a negative feedback regulatory pathway modulating endothelial cell inflammation [25]. Our study implied a relationship between miR-146b-3p and thrombosis, and the mechanism of the negative feedback effect of miR-146b-3p on inflammation needs further study.

There are still some limitations in our study. We only detected COX-2 expression and p38MAPK phosphorylation after we transfected cells with miRNA-146b-3p or its inhibitor. Based on the existing data, we suggested that miRNA-146b-3p could be regulated by inflammatory factors and play a key role in regulating MAPK/COX-2 expression. However, we did not treat the transfected cells with Ang II or TNF-*α* again to observe MAPK/COX-2 expression. Due to the design limitation, we only studied the relationship between and among miRNA-146b-3p, p38MAPK phosphorylation, and COX-2 expression with inflammatory factor treatment. We did not detect the status of other miR-146b target genes either; therefore, we got limited findings unfortunately. Further studies need to be designed to clarify the signaling pathway. In the present study, miR-146b-3p expression levels were detected at 2 h by Ang II treatment and at 4 h by TNF-*α* treatment; further experiments could be done to detect the expression of miR-146b-3p at different time points after inflammatory factor treatment.

In summary, the major finding of this study is that the expression of miR-146b-3p is closely related to inflammation and thrombosis. Inflammatory factors may induce thrombosis through the miR-146b-3p/P38MAPK/COX-2 pathway. However, the mechanism of the negative feedback effect of miR-146b-3p on inflammation needs further study.

## Data Availability

Readers can email the authors (lunysu85@163.com) for accessing the data used in the manuscript.
